# Cognitive decision-making strategies in a haptic angle sorting task: a behavioral experimental study

**DOI:** 10.3389/fnins.2026.1733229

**Published:** 2026-03-30

**Authors:** Yulong Liu, Yali Xiang, Huazhi Li, Mengni Zhou, Qingqing Li, Hongtao Yu, Yoshimichi Ejima, Takahashi Satoshi, Jiajia Yang, Jinglong Wu

**Affiliations:** 1 Key Laboratory of Intelligent Monitoring on Navigation Safety, Hunan University of Information Technology, Changsha, China; 2 Liaoning Normal University, Dalian, Liaoning, China; 3 College of Software, Taiyuan University of Technology, Taiyuan, China; 4 School of Education, Wenzhou University, Wenzhou, China; 5 Cognitive Neuroscience Lab, Graduate School of Interdisciplinary Science and Engineering in Health Systems Okayama University, Okayama, Japan

**Keywords:** angle discrimination, cognitive flexibility, decision-making strategy, efficiency-precision trade-off, haptic perception, sorting tasks

## Abstract

Haptic angle discrimination is an important paradigm for studying human perceptual decision-making. Traditional research has mostly focused on the measurement of perceptual sensitivity (such as thresholds), while neglecting the crucial role of cognitive strategies during task execution. This study aims to explore how different cognitive decision-making strategies affect behavioral performance (discrimination accuracy and decision-making efficiency) in the task of sorting from the tactile perspective. We recruited 18 healthy subjects and asked them to complete the same Angle ranking task using two strategies respectively: (1) the “successive minimum Angle” strategy stipulated in the experiment; (2) the optimal strategy chosen by the subjects themselves. We fitted the perceived uncertainty parameter (*σ*) from the ranking results through the maximum likelihood estimation method and used it as an indicator of discrimination accuracy. At the same time, we recorded the number of adjustments during the decision-making process as an efficiency indicator. The results show that, compared with the fixed “successive minimum” strategy, when the self-selected strategy is adopted, the decision-making efficiency (number of adjustments) of the subjects is significantly improved (*p* < 0.001), while there is no significant loss in the discrimination accuracy (Sigma value) (*p* > 0.05). Further qualitative analysis revealed that the vast majority of the subjects (14/18) spontaneously adopted the advanced cognitive strategy of “double-boundary—limit transition,” which maximizes efficiency by dynamically managing the working memory load and the number of comparisons. This study quantified for the first time the optimization effect of strategy flexibility on the decision-making process in the tactile sequencing task, revealing that the human cognitive system can adaptively select strategies according to task requirements to achieve the best trade-off between efficiency and accuracy. This discovery holds significant theoretical importance for constructing more comprehensive perceptual decision-making computing models and understanding human adaptive behaviors in complex environments.

## 1 Introduction

Perceptual Decision-Making is the core cognitive process that connects sensory input with behavioral output. In daily life, we constantly make judgments and choices based on sensory information, such as judging the texture, shape or size of objects by touch (Ryan et al., 2021; Rezaul Karim et al., 2022; Sharma et al., 2022; Hidaka et al., 2024; Lorenz and Kucker, 2025). Tactile angle discrimination, as a classic psychophysical task, is widely used to study the mechanism of tactile perception (Wu et al., 2010, 2020; Yang et al., 2010; Wang et al., 2019, 2020; Zhang et al., 2020; Liu et al., 2021). In this paradigm, researchers typically focus on the human perceptual limit, namely the so-called “Difference Threshold” or “Just Noticeable Difference” (JND), which reflects the intrinsic noise level or resolution of the perceptual system (Frahm and Gervasio, 2021; Li et al., 2022).

However, a key point that is often overlooked is that the overall behavioral performance of completing a perceptual task (such as sorting) does not only depend on the underlying perceptual sensitivity, but is also largely regulated by the high-level Cognitive Strategy (Liu et al., 2021). For example, when asked to sort multiple tactile stimuli by Angle size, subjects can adopt various strategies: they can repeatedly compare all stimulus pairs (exhaustive comparison), or they can use strategies such as “selection sort” or “bubble sort,” solving only one maximum or minimum value at a time (Suleiman Al-Kharabsheh et al., 2013). Each strategy shows significant differences in cognitive load, working memory requirements, and the number of comparisons needed, which directly affect the efficiency of task completion and the ultimate achievable accuracy (Tulsky et al., 2014; Dann et al., 2023).

“Speed-Accuracy Trade-off” (SAT) is a fundamental principle in decision science (Vandierendonck, 2021). Usually, the pursuit of faster speed (higher efficiency) may come at the expense of accuracy (precision), and vice versa. However, experienced decision-makers can often significantly enhance efficiency while maintaining or even improving accuracy by adopting smarter strategies (Derosiere et al., 2021). This means that studying the strategy itself, rather than just the final perceptual output, is crucial for a comprehensive understanding of human decision-making behavior.

This study designed an innovative behavioral experiment to directly explore the role of cognitive strategies in the task of haptic Angle sequencing. We require the same group of subjects to complete the same task under two conditions: (1) Strategy constraint: It is stipulated that the subjects use the strategy of “selecting the smallest Angle successively” (an algorithm similar to selection sort) to fix their decision-making path; (2) Strategy freedom condition: Participants are allowed to adopt any strategy they consider the most efficient to complete the ranking and report the strategy they used afterwards. We infer the Sigma value representing the perceived uncertainty from the ranking results of each attempt through a computational model (maximum likelihood estimation), which is used as a quantitative indicator of discrimination accuracy (Liu et al., 2021). Meanwhile, we recorded the number of times the subjects adjusted the stimulus sequence before making the final decision as an indicator of decision-making efficiency.

The core innovation points and hypotheses of this study lie in the following four aspects. Firstly, this study innovatively quantified the strategy effect, that is, we took the strategy itself as an independent variable and quantitatively studied its independent impact on decision-making efficiency and high-level perception accuracy (rather than the low-level threshold). Secondly, the hypothesis of “strategic flexibility” is proposed; We assume that the human cognitive system has Strategic Flexibility and can adaptively select the optimal strategy to optimize behavioral performance. We predict that under the free strategy condition, the subjects can significantly reduce the number of adjustments required for decision-making (i.e., improve efficiency) by adopting more efficient strategies while maintaining the accuracy (Sigma value) without a significant decline. Furthermore, we expect that the strategies spontaneously adopted by the subjects will not merely be simple algorithms, but will combine the characteristics of perceptual tasks (such as sequential processing of tactile information and working memory limitations) to form more complex and efficient Meta-strategies. Finally, we adopt the trial-by-trial behavior output (ranking sequence) to fit the perceptual uncertainty, providing a new methodological idea for evaluating perceptual accuracy in complex tasks. This research not only helps to deepen our understanding of the cognitive factors in tactile perception decision-making, but its findings will also provide inspirations from human cognition for the design of efficient decision-making algorithms in fields such as artificial intelligence and human-computer interaction.

## 2 Materials and methods

### 2.1 Participants

**Figure 1:**
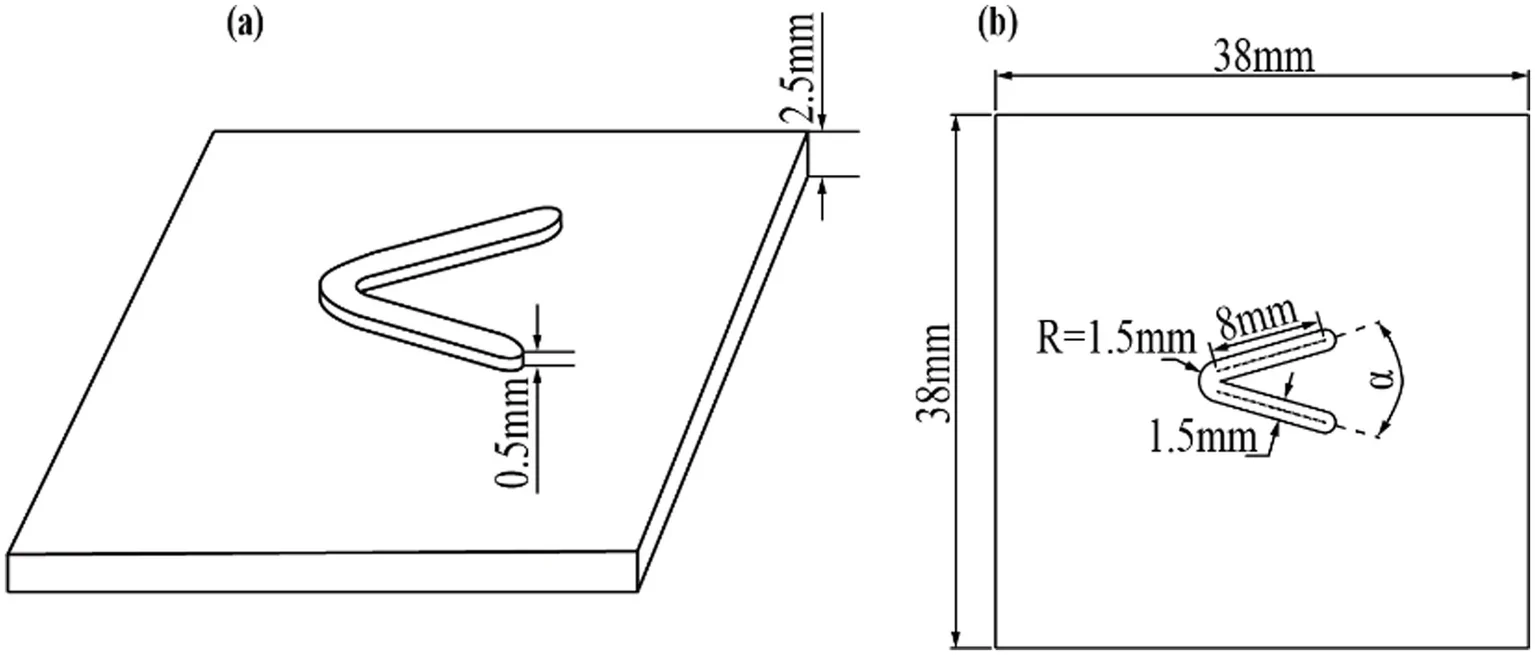
The design of a haptic pattern. These raised-angle patterns which are part of the top of the angle stimulation block touch the index finger.

**Figure 2:**
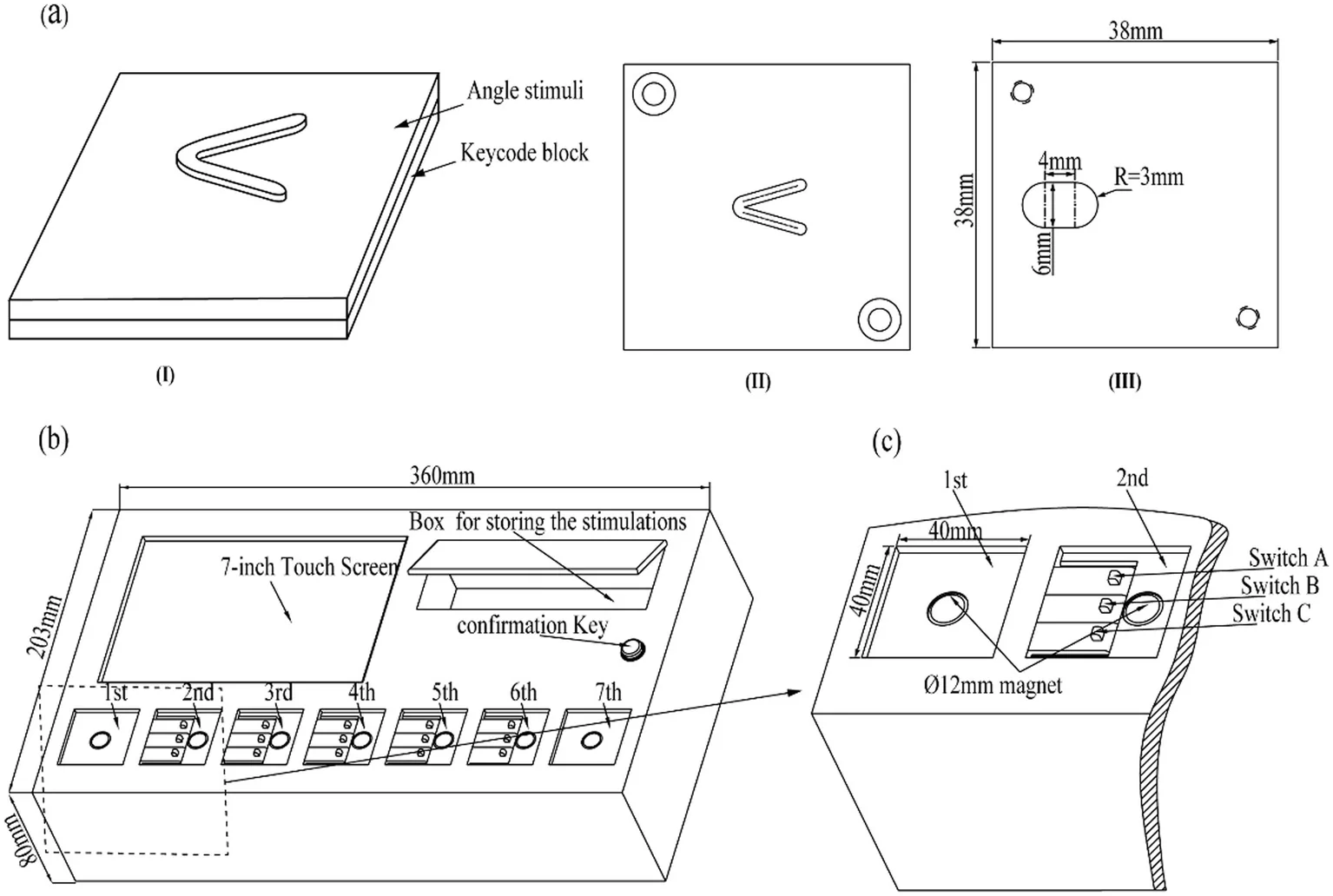
The configuration of the haptic angle sorting device. **(a)** Design of the haptic pattern (I), which includes the angle stimulus at the front (II) and keycode base at the back (III); **(b)** Interface design of the haptic angle device; and **(c)** Design of the haptic angle stimulus position in a fixed position and a sequence position. The actions of three microswitches (switches A, B, and C) at the sorting positions, along with the unique trigger codes for each stimulus, are used to accurately identify the stimulus. When placing the angle stimulation module, which is composed of the angle stimulation and iron base block, the stimulus module at each position is attracted by a 12 mm-diameter magnet and triggers the microswitch in the groove.

In our previous studies, stimuli of two-dimensional (2-D) raised-line drawings were used to explore the touch perception process (Picard et al., 2010; Wu et al., 2010; Yang et al., 2010; Lebaz et al., 2012; Zhang et al., 2019, 2020). The accuracy of 2-D raised-line drawings is amenable to control when being designed, and the drawings can remove the information of real objects. Therefore, we designed a 2-D raised-angle stimulus for the sorting test, where the angle difference of the 2-D raised-angle is 2°, as shown in Figure 1. The sorting experiment used seven angle stimuli with a 2-degree angular difference, and the angular stimuli were distributed as 20°, 22°, 24°, 26°, 28°, 30°, and 32°. where the smallest angle of 20 degrees and the largest angle of 32 degrees were used as the reference angles. After the subjects confirmed the magnitude of the angular stimulus through touch, they were placed from left to right in sequence from small to large at the second to sixth positions of the device (the first and seventh positions were, respectively, placed with the minimum and maximum reference stimuli), as shown in Figure 2. Three microswitches (switch A, B, and C) are arranged in a group in each position from the grooves of the second to sixth positions to identify each angle stimulus. The five angular stimuli correspond to five button codes (000, 001, 011, 100, and 110), each corresponding to the angular stimulus value and its output binary data value (Liu et al., 2021).

### 2.2 Ethics statement

The study was conducted in accordance with the principles of the Declaration of Helsinki and was approved by the Institutional Review Board at Okayama University. Written informed consent was obtained from all participants prior to their involvement in the study. None of the participants had prior experience with the haptic angle sorting test.

### 2.3 Procedure

In the haptic angle sorting task, the experiment included a total of 7 angle stimuli. In this study, the difference between adjacent angle stimuli is 2°, which includes a minimum reference angle, a maximum reference angle, and five different angles between these two reference angles (Wijntjes and Kappers, 2007; Wu et al., 2010; Yang et al., 2010; Liu et al., 2021). All angular stimuli are placed horizontally, with the vertex facing right and the two sides facing left. At the beginning of the experiment, the minimum reference angle and the maximum reference angle were fixed at the first and seventh positions respectively, providing the subjects with a clear reference framework and effectively reducing the difficulty of the Angle sorting task. The remaining five angles of stimulation were presented in a random order at the 2nd to 6th positions all at once. Throughout the entire experiment, the subjects were allowed to touch the reference angles of the first and seventh positions, but were not permitted to move their positions. The task of the subjects was to arrange the five Angle stimuli from the second to the sixth in ascending order. The direction of stimulation at each Angle remains unchanged, but the subject can adjust its relative position between the 2nd and 6th positions an unlimited number of times. The subjects need to complete the experiment while wearing eye masks. Before the experiment, adjust the height of the seat and the table to ensure that the subjects’ arms can operate the equipment comfortably. After each adjustment of the stimulus position, the system will record the current sorting status and ask the subject to confirm the Angle sequence from left to right. According to the pre-experiment assessment, all participants can submit the final results after approximately 3 to 5 rounds of sorting and confirmation. Each Angle sorting task (1 trial) only takes about 3 to 5 min to complete (Liu et al., 2021).

The subjects need to complete the sorting task under the following two strategic conditions:

Minimum Angle Strategy Condition (MAS): Under this condition, the subjects are required to strictly follow the following fixed procedures for operation:

a) Select the smallest of the five angles from the 2nd to the 6th position and adjust it to the 2nd position;
b) Select the smallest one from the remaining four angles again and adjust it to the third position;
c) Repeat this process until all angles are arranged in ascending order.

Each time the order is selected and adjusted; the system will record the current sorting status. When the subject confirms the final sequence, press the confirmation button to complete the test.

Free Strategy Condition (FS): Under this condition, the subjects are only required to complete the sorting task “by using what they consider to be the fastest and most accurate method.” The subjects can freely explore the stimuli, make any number of pairwise comparisons, and adjust the current ranking assumptions at any time. Each time the order is adjusted, the system will also record the sorting status. When the subject confirms the final sequence, press the confirmation button to complete the test.

To avoid mutual interference between the learning effect of strategies, the time interval for each subject to complete the two strategy experiments was 2 weeks. All the participants adopted a balanced design in the order of two strategies: two people formed a group, with one person first performing Strategy 1 (the minimum angle strategy) and then Strategy 2 (the free strategy), while the other person adopted the reverse order. This design effectively controls the possible deviations caused by the experimental sequence. The equations should be inserted in editable format from the equation editor.

### 2.4 Data processing and analysis

For each trail, we separately recorded the sequence of the five angle stimuli (positions 2–6) after each adjustment, as well as the total number of adjustments made before final confirmation. Based on the final sorted sequence, we used maximum likelihood estimation (MLE) to calculate the Discrimination Index (DI, denoted as *σ*), which represents perceptual sensitivity in discrimination (Liu et al., 2021).

Let the five angle stimuli be denoted as $S=\{s_{1},s_{2},s_{3},s_{4},s_{5}\}$ , corresponding to the physical angles 22°, 24°, 26°, 28°, and 30° respectively. In a correct ascending order, the stimulus at position *j* (where *j* = 2,3,4,5,6 corresponds to the 2nd to 6th positions in the apparatus) should be $s_{j-1}$ . Let the actual sorted sequence be $X=[x_{2},x_{3},x_{4},x_{5},x_{6}]$ , where $x_{j}\in S$ represents the stimulus placed at position *j*. We assume that each stimulus $s_{i}$ has an internal perceptual representation $p_{i}$ that follows a Gaussian distribution centered on its physical value, with a common standard deviation *σ* (the DI parameter to be estimated) (Lee and O’Mahony, 2004; Kim et al., 2006; Liu et al., 2021): $p_{i}∼N(\theta_{i}-\sigma^{2})$ .where $\theta_{i}$ is the physical angle value of stimulus $s_{i}$ . When a subject places stimulus $x_{j}$ at position *j*, we assume this decision is based on comparing the perceptual representations of all remaining stimuli. The probability that stimulus $s_{i}$ is chosen for position *j* given the set of remaining stimuli R is:


$$P_{j}(s_{i}|R,\sigma )=\frac{\phi (\frac{\theta_{i}-\mu_{j}}{\sigma })}{\sum_{s_{k}\in R}\phi (\frac{\theta_{k}-\mu_{j}}{\sigma })}$$ (1)

Where *ϕ* is the probability density function of the standard normal distribution (Equation 1), and $\mu_{j}$ is the expected perceptual value at position *j* (for position 2, this would be near 22°; for position 6, near 30°). For positions 2 to 6, we set $\mu_{j}$ to the physical angle value that should correctly occupy that position (i.e., $\mu_{2}=22^{{}^{\circ}},\mu_{3}=26^{{}^{\circ}},\mu_{4}=24^{{}^{\circ}},\mu_{5}=28^{{}^{\circ}},\mu_{6}=30^{{}^{\circ}}$ ).

The likelihood function for the entire sorted sequence X is constructed as the product of probabilities at each position, accounting for the fact that stimuli are selected without replacement:


$$L(\sigma |X)=\prod_{j=2}^{6}P_{j}(x_{j}|R_{j},\sigma )$$ (2)

where $R_{2}=S$ (all five stimuli available for position 2), $R_{2}=S\backslash \{x_{2}\}$ , and so on (Equation 2). We then find the σ value that maximizes this likelihood function using numerical optimization. A smaller *σ* indicates higher discrimination accuracy (perceptual representations are more tightly clustered around true values), while a larger *σ* indicates poorer discrimination ability (Liu et al., 2021).

Decision-making efficiency was operationalized as the total number of adjustments made by the subject before final confirmation. Each adjustment represents a complete cognitive operation including tactile comparison, decision-making, and motor execution. A higher number of adjustments indicates lower superficial efficiency, but may reflect deeper cognitive processing. To compare efficiency and accuracy across strategies while accounting for individual differences, we computed normalized indices. For each subject under each condition, we defined:

- Efficiency index (E) = 1/ (average number of adjustments);
- Accuracy index (A) = 1/ (average *σ* value);

These indices were normalized using min-max normalization across all subjects and both conditions:


$$E_{\mathrm{norm}}=\frac{E-E_{\min}}{E_{\max}-E_{\min}}$$ (3)

$$A_{\mathrm{norm}}=\frac{A-A_{\min}}{A_{\max}-A_{\min}}$$ (4)

where $E_{\min}$ , $E_{\max}$ , $A_{\min}$ , $A_{\max}$ are the minimum and maximum values observed across all measurements (Equation 3 and 4). This transformation yields indices ranging from 0 to 1, with higher values representing greater efficiency or accuracy.

**Figure 3:**
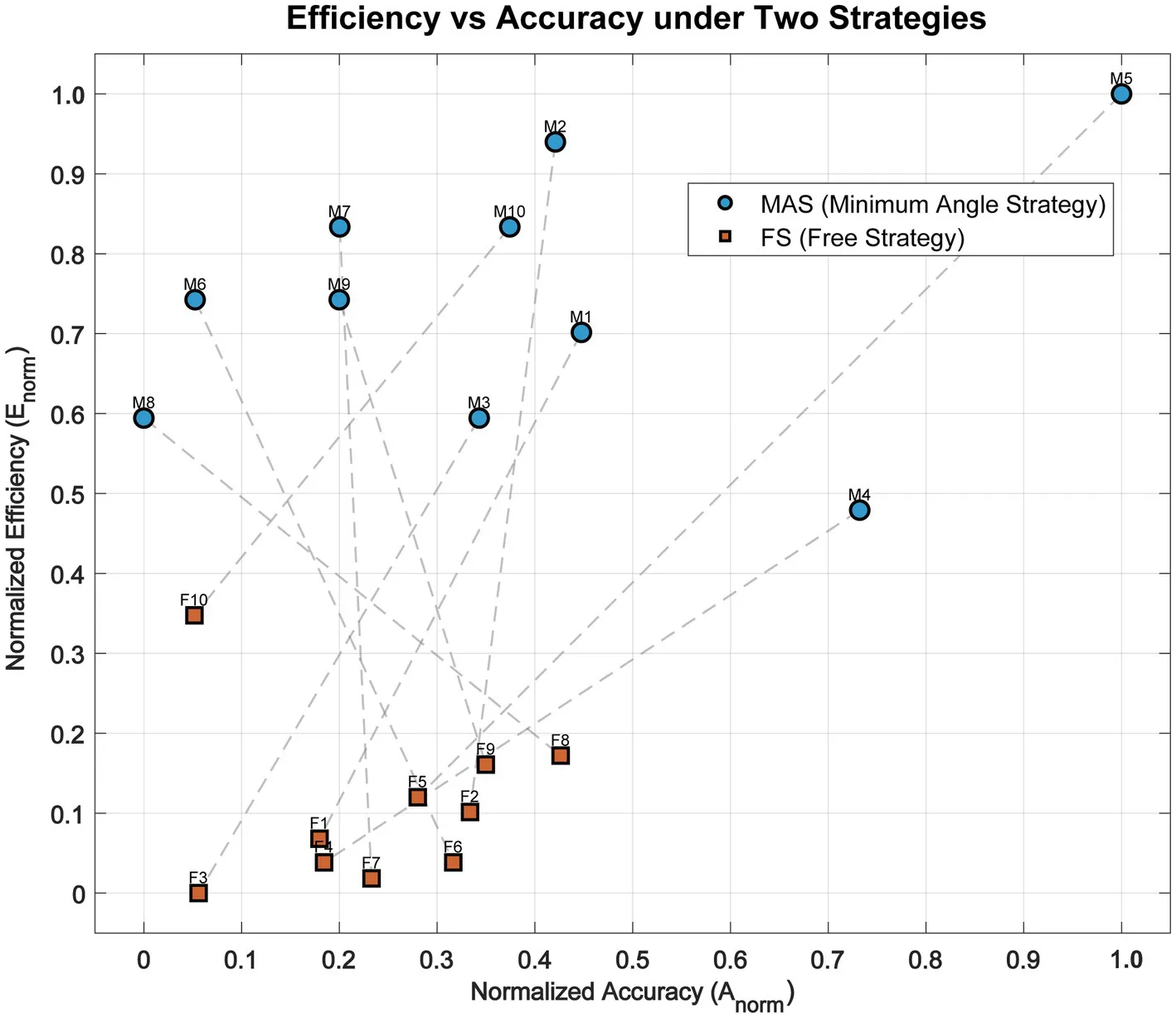
The ternary phase diagram reflecting the relationship among cognitive intensity, efficiency, and accuracy. Points closer to the “Efficiency” vertex represent profiles dominated by high efficiency (few adjustments), points closer to “Accuracy” represent profiles dominated by high accuracy (low *σ*), and points closer to “Cognitive Intensity” represent profiles where both efficiency and accuracy are high, resulting in a high product term. The position of a point in the graph is determined by the relative magnitudes of the three dimensions; the closer a point is to a vertex, the more dominant that dimension is. The horizontal axis primarily reflects the trade-off between efficiency and accuracy, while the vertical axis primarily reflects the strength of cognitive ability.

To visualize the relationship between efficiency and accuracy under the two strategy conditions, we constructed a two-dimensional scatter plot of $E_{\mathrm{norm}}$ versus $A_{\mathrm{norm}}$ (Figure 3). This plot allows direct inspection of the trade-off (or lack thereof) between the two dimensions and reveals how the free strategy (FS) shifts the distribution relative to the fixed minimum-angle strategy (MAS).

### 2.5 Statistics

We conducted statistical analysis using SPSS Statistics (IBM). Firstly, we conducted Repeated Measures ANOVA for the Sigma value and the number of adjustments respectively, with the policy condition (MAS vs. FS) as the within-subject factor. When the ANOVA results show a significant main effect, a *post hoc* paired t-test is conducted. In addition, we calculated the Pearson correlation coefficients of Sigma and the number of adjustments under the two conditions to test whether there is a trade-off relationship between efficiency and accuracy. The significance level *α* was set at 0.05. Bonferroni correction was performed on the *p*-values of all post hoc tests. It also conducts qualitative data analysis. Summarize and classify the strategies reported orally by the subjects, and calculate the usage frequency of each type of strategy.

## 3 Result

### 3.1 Decision-making efficiency (number of adjustments)

Repeated measures analysis of variance showed that the main effect of strategy conditions on the number of adjustments was extremely significant (*F* (1, 17) = 185.3, *p* < 0.001, η^2^ = 0.91). Contrary to our initial hypothesis, subjects made significantly more adjustments under the FS condition (M = 3.89, SD = 0.32) compared to the MAS condition (M = 1.72, SD = 0.15). This indicates that when given freedom to choose their own strategy, subjects did not minimize behavioural steps, but instead engaged in more extensive exploration and intermediate comparisons (Table 1).

**Table 1:** The average number of adjustments and discrimination index under the two strategy conditions. (M ± SEM).

Indicator	Minimum angle strategy (MAS)	Free strategy (FS)	Statistical results
Number of adjustments	1.72 ± 0.15	3.89 ± 0.32	*F*(1,17) = 185.3, *p* < 0.001
Discrimination index (DI)	1.08 ± 0.15	1.22 ± 0.14	F(1,17) = 1.52, *p* = 0.234

### 3.2 The accuracy of DI (σ value)

No significant main effect of strategy condition was found for *σ* values (*F*(1, 17) = 1.52, *p* = 0.234, η^2^ = 0.08). The average σ under MAS condition (M = 1.08, SD = 0.15) was not significantly different from that under FS condition (M = 1.22, SD = 0.14). This suggests that despite making more adjustments, subjects achieved comparable final accuracy under both conditions.

### 3.3 The relationship between efficiency and accuracy

Figure 3 presents the normalized efficiency $\left(E_{\mathrm{norm}}\right)$ and accuracy $\left(A_{\mathrm{norm}}\right)$ for each subject under both strategies. Points in the upper-right quadrant indicate high efficiency and high accuracy. Under the MAS condition, data points cluster in a region of moderate efficiency and moderate accuracy, with relatively little dispersion. Under the FS condition, points are more widely scattered: some subjects achieved higher efficiency, some higher accuracy, and many shifted toward the upper-right quadrant, indicating simultaneous gains in both dimensions. A paired comparison of the Euclidean distance from the origin $\left(\sqrt{E_{\mathrm{norm}}^{2}+A_{\mathrm{norm}}^{2}}\right)$ revealed a significant increase under FS (*t*(17) = 3.12, *p* = 0.006), confirming that free strategy allowed subjects to reach a more favorable efficiency-accuracy combination overall.

**Figure 4:**
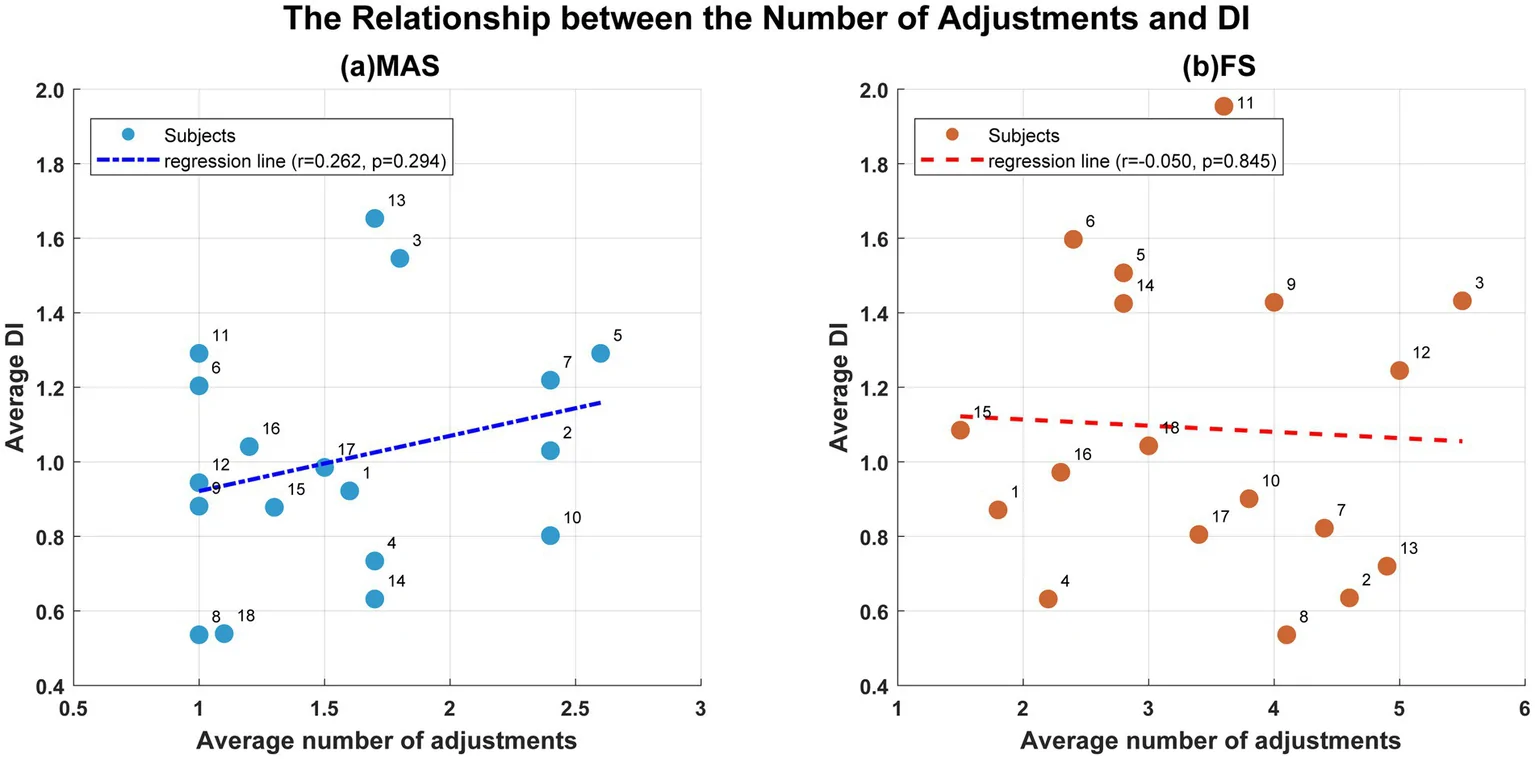
The correlation analysis of the number of adjustments and DI under two sorting strategic conditions. Scatter plots showing the relationship between the number of adjustments and the discrimination index (σ) under **(a)** minimum angle strategy (MAS) and **(b)** free strategy (FS). Each point represents one participant. Regression lines and Pearson correlation coefficients (*r*) are shown; neither correlation reached statistical significance (MAS: *p* = 0.294; FS: *p* = 0.845). The near-zero correlation under FS indicates that the increased number of adjustments was not associated with systematic changes in discrimination accuracy.

In addition, Pearson correlation analysis showed no significant correlation between number of adjustments and σ value under either condition (MAS: r = 0.262, *p* = 0.294; FS: r = −0.050, *p* = 0.845), as shown in Figure 4. Crucially, the dissociation between increased adjustments and stable accuracy across conditions challenges the traditional speed-accuracy trade-off framework, suggesting that the additional adjustments under FS serve a cognitive optimization function rather than reflecting inefficiency.

### 3.4 The quality of strategic choice

Summarizing the strategies of the subjects’ oral reports, it was found that there are three main types:

1. Double-boundary-Limit transition strategy (N = 14): This is the most mainstream strategy. The subjects reported their strategy as follows: “First, quickly explore the five angles to identify the two smallest and largest ‘boundary’ stimuli in the sensation,” “First, find the smallest and largest ones and place them at both ends, and then deal with the middle three,” “By determining the two poles, quickly narrow down the uncertainty range of the middle part.” This is an extremely efficient meta-strategy that decomposes a global sorting problem of five elements into smaller and more manageable local sorting problems by prioritizing the anchors of the sorting sequence.
2. Overall comparison after full memorization (*N* = 2): Two subjects reported attempting to store all Angle information in their working memory and then conduct a “mental ranking” in their minds. This strategy has extremely high requirements for working memory load.
3. Selection of the smallest strategy (*N* = 1): One subject still adhered to the “successive selection of the smallest” strategy under free conditions, which was the same as the MAS condition. Another subject reported the use of the “single boundary” (only finding the minimum) strategy.

The qualitative data are highly consistent with the quantitative data. The subjects who adopted the “double-boundary-limit transformation” strategy generally had a higher number of adjustments under the FS condition than under the MAS condition, but the σ value remained stable. This proves that the additional number of adjustments they made was not an invalid operation, but was used to implement a more complex but more robust decision-making plan.

## 4 Discussion

This study, through ingenious experimental design, separated the perceptual precision component and the cognitive strategy component in the tactile Angle sequencing task, and achieved findings that were partially contrary to the initial hypothesis but more enlightening. Our results clearly demonstrate that human cognitive decision-making systems possess a high degree of strategic flexibility and are capable of adopting seemingly inefficient but actually more robust complex strategies to optimize the final overall performance (Laureiro-Martínez and Brusoni, 2018; Cherry et al., 2021).

### 4.1 Re-examining the “efficiency-accuracy trade-off”

The most striking finding is that the FS condition did not reduce the number of adjustments; instead, it significantly increased them. This directly challenges the simple assumption that “fewer steps mean higher efficiency.” Under MAS conditions, the low number of adjustments is a result of algorithmic compulsion, and its cognitive cost may be transferred elsewhere, such as the comparison load required for each “minimum choice” decision. In contrast, the increased number of adjustments under the FS condition essentially represents an investment in a better decision-making framework—the “double-boundary limit transition” strategy. The subjects voluntarily sacrificed superficial, local efficiency (more adjustments) in exchange for global, overall benefits.

This interpretation is supported by the two-dimensional efficiency-accuracy plot (Figure 3). Under FS, subjects achieved, on average, a more favorable combination of normalized efficiency and accuracy, with several individuals reaching points that dominate their MAS performance in both dimensions. The lack of a negative correlation between adjustments and accuracy under FS (Figure 4, r = −0.050) further indicates that the additional steps were not merely “wasted” but contributed to maintaining or even improving final accuracy. This pattern reflects a deeper concept of cognitive efficiency-not merely minimizing external behavioral steps, but optimally allocating internal cognitive resources (working memory, comparison load, decision certainty) to achieve robust performance.

On the contrary, the increased number of adjustments under the FS condition is essentially an investment in a better decision-making framework - the “double-boundary - limit transformation” strategy. The subjects voluntarily sacrificed superficial and local efficiency (more adjustments) in exchange for global and overall benefits (Förster and Higgins, 2005):

1. Reduce decision-making uncertainty (Lipshitz and Strauss, 1997; Danczak and Lea, 2017): Prioritize the determination of the most extreme (minimum and maximum) stimuli, providing a stable anchor point for the entire sequence. These two stimuli usually have the greatest perceived difference, the highest signal-to-noise ratio in discrimination, and the lowest probability of error.
2. Simplify the remaining problems (Ho et al., 2022): Once the beginning and end of the sequence are fixed, the sorting problem of the middle part is simplified from 5 out of 5 to 3 out of 3, greatly reducing the complexity and comparison range of subsequent decisions.
3. Reduce working memory load (Dalton et al., 2009; Sweller, 2024): There is no need to remember all stimuli simultaneously and sort them. Only local adjustments need to be made within a determined framework, which reduces cognitive load.

Therefore, what this study reveals is a deeper concept of Efficiency - Cognitive Efficiency. It is not only about external behavioral steps, but also about the internal allocation of cognitive resources and the management of computational complexity (Rypma and Prabhakaran, 2009; Hoffman, 2012). The FS strategy achieves an optimized form without loss of accuracy by adding controllable and purposeful steps in exchange for an improvement in decision reliability and a reduction in cognitive load (Payne and Bettman, 2002; Rachmad, 2022), as shown in Figure 3. This is an intelligent trade-off of “exchanging process for precision and stability.”

### 4.2 The cognitive mechanism of the “double boundary-limit conversion” strategy

This study reports for the first time the universality of the “double-boundary - limit transition” strategy (Adaptive Strategy) in the tactile sorting task. This strategy reflects several characteristics of advanced human cognitive functions:

1. Hierarchical problem-solving: Capable of breaking down complex problems into sub-problems and prioritizing the resolution of key sub-problems (identifying boundaries) (Chen, 2002).
2. Active cognitive control: Not passively following the stimulus input, but actively generating and testing hypotheses (“Is this the smallest? “) Is that the biggest one? And based on this, plan the exploration sequence (Pezzulo, 2012).
3. Utilizing perceptual ecology: In natural environments, extreme values (such as maximum, minimum, fastest, and slowest) often carry the most crucial information. The human perceptual system may have evolved a tendency to be more sensitive to or prioritize extreme values (Rust and Cohen, 2022; Shevlin et al., 2022).

This strategy coincides with the “Divide and Conquer” algorithmic idea in computer science (Smith, 1985), demonstrating the sophistication of the human brain as a biological computer in handling optimization problems.

### 4.3 The significance and implications of the research

This study emphasizes the importance of incorporating strategic factors in touch perceptual decision-making research. Traditional psychophysical measurements (such as thresholds) may confuse perceptual abilities with cognitive strategies. The future perception model needs to be an integrated “perception-cognition” model, taking strategy selection as a core parameter.

Furthermore, the σ value estimation method based on MLE that we adopt provides an effective tool for inferring internal perceptual states from complex behavioral data (Liu et al., 2021), particularly in experimental paradigms where confidence reports are not directly available.

1. Human-Computer Interaction and Usability Engineering: When designing interfaces that require users to make multiple comparisons and decisions (such as product selection and parameter adjustment), the “double boundary” strategy can be referred to, prioritizing the guidance of users to determine the two extremes of their preference range, thereby enhancing the decision-making experience and efficiency.
2. Artificial Intelligence: The current decision-making process of AI often lacks such hierarchical and strategic planning. This research provides valuable insights for developing more biologically plausible and computationally efficient decision-making algorithms inspired by human perceptual-cognitive integration.
3. Neurorehabilitation: For individuals with impaired tactile perception, targeted cognitive strategy training-such as instruction in effective exploration techniques-may serve as a compensatory approach to mitigate perceptual deficits and improve performance in activities of daily living (von Bastian et al., 2022a, 2022b).

### 4.4 Limitations and future research directions

Lashley (1930) this study has several limitations that should be acknowledged and addressed in future research. First, the sample size (*N* = 18), while sufficient to detect large effects on behavioral measures, may limit the generalizability of our findings and the statistical power for detecting more subtle individual differences in strategy use. Future studies with larger samples could employ cluster analysis or latent class modeling to identify distinct strategy profiles and their correlates with individual differences in cognitive abilities (e.g., working memory capacity, tactile acuity). Second, the relative simplicity of the task—sorting only five stimuli with a constant 2° difference—may have constrained the range of strategies observed. While this design was chosen to isolate perceptual and strategic factors, tasks with higher cognitive load (e.g., more stimuli, variable step sizes, or time pressure) might reveal different strategy dynamics or more pronounced efficiency-accuracy trade-offs. Future studies should systematically vary task complexity to examine how strategy selection adapts to increasing cognitive demands. Third, the task was unimodal (tactile only) and used artificial raised-line angles rather than naturalistic objects. Real-world haptic perception typically involves multiple cues (texture, compliance, temperature) and active exploration. Whether the double-boundary strategy generalizes to multimodal or naturalistic contexts remains an open question. Cross-modal comparisons (e.g., visual vs. haptic sorting) could reveal modality-specific or modality-general aspects of strategy selection. Fourth, our behavioral measures and verbal reports provide only indirect access to cognitive processes. Future studies employing neuroimaging (fMRI, EEG) or eye-tracking (in visual analogs) could directly test neural predictions, such as increased prefrontal involvement during boundary-setting phases of the double-boundary strategy. Additionally, computational modeling of the decision process at the level of individual comparisons could provide more fine-grained insights into the underlying cognitive mechanisms. Finally, the counterbalancing interval of 2 weeks between conditions, while sufficient to minimize carryover effects, may have introduced uncontrolled variability in tactile sensitivity or motivation. Future studies could employ within-session manipulations with different stimulus sets to better control for state-dependent factors.

Despite these limitations, the present study provides robust evidence for strategic flexibility in haptic decision-making and offers a methodological framework for investigating perception-cognition interactions in complex tasks.

## 5 Conclusion

This study, through a behavioral experiment on tactile angle sequencing, provides robust evidence for the critical role of cognitive decision-making strategies in shaping behavioral performance. Humans exhibit remarkable strategic flexibility in perceptual decision-making: rather than adhering to algorithms with only one strategy (i.e., minimal procedural steps), they adaptively select higher-level meta-strategies in response to task demands. The “double-boundary limit transformation” strategy, voluntarily adopted by the majority of participants, although entailing increased behavioral steps (i.e., adjustment times), effectively enhances decision accuracy by reducing decision uncertainty, simplifying task complexity, and alleviating working memory load—thereby achieving a deeper level of cognitive optimization. These findings challenge the traditional efficiency-precision trade-off theory by proposing an alternative model of “exchanging process complexity for precision stability,” in which efficiency is redefined as the optimal allocation of cognitive resources rather than mere minimization of observable actions. In conclusion, this research highlights the high degree of adaptability and intelligence inherent in the human cognitive system when confronting complex perceptual tasks, offering a novel and significant perspective on perception-cognition interactions.

## Data Availability

The original contributions presented in the study are included in the article/supplementary material, further inquiries can be directed to the corresponding author.
